# Patterns of Extrathoracic Metastases in Different Histological Types of Lung Cancer

**DOI:** 10.3389/fonc.2020.00715

**Published:** 2020-05-19

**Authors:** Xuan Wang, Zheng Wang, Junjie Pan, Zhou-Yi Lu, Dong Xu, Hui-Jun Zhang, Shao-Hua Wang, Da-Yu Huang, Xiao-Feng Chen

**Affiliations:** ^1^Department of Thoracic Surgery, Huashan Hospital, Fudan University, Shanghai, China; ^2^Comprehensive Breast Health Center, Ruijin Hospital, Shanghai Jiao Tong University School of Medicine, Shanghai, China; ^3^Department of General Surgery, Huashan Hospital, Cancer Metastasis Institute, Fudan University, Shanghai, China

**Keywords:** lung cancer, metastasis, histological type, survival, pattern

## Abstract

Lung cancer is the leading cause of cancer-related deaths mainly attributable to metastasis, especially extrathoracic metastasis. This large-cohort research is aimed to explore metastatic profiles in different histological types of lung cancer, as well as to assess clinicopathological and survival significance of diverse metastatic lesions. Lung cancer cases were extracted and enrolled from the Surveillance, Epidemiology, and End Results (SEER) database. χ^2^-tests were conducted to make comparisons of metastatic distribution among different histological types and odds ratios were calculated to analyze co-occurrence relationships between different metastatic lesions. Kaplan–Meier methods were performed to analyze survival outcomes according to different metastatic sites and Cox regression models were conducted to identify independent prognostic factors. In total, we included 159,241 lung cancer cases with detailed metastatic status and complete follow-up information. In order to understand their metastatic patterns, we elucidated the following points in this research: (1) Comparing the frequencies of different metastatic lesions in different histological types. The frequency of bone metastasis was highest in adenocarcinoma, squamous cell carcinoma, LCLC and NSCLC/NOS, while liver was the most common metastatic site in SCLC. (2) Elaborating the tendency of combined metastases. Bi-site metastases occurred more common than tri-site and tetra-site metastases. And several metastatic sites, such as bone and liver, intended to co-metastasize preferentially. (3) Clarifying the prognostic significance of single-site and bi-site metastases. All single-site metastases were independent prognostic factors and co-metastases ended up with even worse survival outcomes. Thus, our findings would be beneficial for research design and clinical practice.

## Introduction

Lung cancer is one of the most common malignancies and the leading cause of cancer-related deaths (1). Every year, 1.8 million new cases are diagnosed and 1.6 million lung cancer related-deaths occur worldwide (2). This fatal neoplasm represents a typical example for which metastatic patients tend to have extraordinary poorer prognosis than non-metastatic cases (3, 4). In spite of the rapid development of novel therapeutic methods, such as epidermal growth factor receptor tyrosine kinase inhibitors (EGFR TKI), anaplastic lymphoma kinase (ALK) inhibitors and immune checkpoint inhibitors, 5-year overall survival remains relatively low mainly attributable to high risk of distant metastasis (5–7).

To date, tumor hallmarks, metastatic patterns and prognostic outcomes differ greatly among different histological types of lung cancer (8). Non-small cell lung cancer (NSCLC), including adenocarcinoma, squamous cell carcinoma, large-cell lung cancer (LCLC) and others that are not otherwise specified (NOS), accounts for more than 80% of all lung cancers (9). As for small-cell lung cancer (SCLC), making up <20% of all histological types, it is the most aggressive form of lung cancer and featured by malignant proliferation and early invasive spread (10, 11).

Tumor, regional lymph node and metastasis (TNM) staging system was universally applied for prognostic prediction and therapeutic guidance. According to the 8th TNM staging by American Joint Committee on Cancer (AJCC), M1a was defined as intrathoracic metastases including contralateral lung nodules, pleural metastases and pericardial effusion, and M1b or M1c were defined as single or multiple extrathoracic metastases (12). Previous researches suggested that patients with extrathoracic metastasis had markedly shortened survival than limited intrathoracic metastasis (13–15). Therefore, it is vital to draw a detailed landscape for patients with extrathoracic metastasis.

However, extrathoracic metastatic patterns of lung cancer and their diversity in different histological types are unclear and need further clarification. And prognostic outcomes of diverse extrathoracic sites need to be investigated. Thus, this retrospective, large-cohort study is aimed to explore metastatic profiles in different histological types of lung cancer, as well as to assess clinicopathological and survival significance of diverse metastatic lesions.

## Methods

### Cohort Population

We performed a retrospective, population-based research by extracting data from the Surveillance, Epidemiology, and End Results (SEER) national database. Cases were included in this research on the basis of the following inclusion and exclusion criteria.

Inclusion criteria: (1) Diagnosis of lung cancer was made pathologically between the year 2010–2014; (2) Lung cancer was the first primary malignancy; (3) Detailed information about metastatic status was complete.

Exclusion criteria: (1) Age under 18 years old; (2) Metastatic status was unknown; (3) Follow-up data was missing; (4) Information about histological type was unknown.

### Statistical Analysis

Descriptive statistics were used to summarize patients' demographic, clinicopathological, and therapeutic variables in different histological subgroups. We conducted χ^2^-tests to make comparisons of metastatic distribution among different histological types. Odds ratios were calculated to analyze co-occurrence relationships between different metastatic lesions. Kaplan–Meier methods were performed to analyze overall survival (OS) and cancer-specific survival (CSS) according to different metastatic sites were conducted to identify independent prognostic factors. Two-sided *P* < 0.05 were defined as statistically significance. We used GraphPad Prism 7 (GraphPad Software, San Diego, CA, USA) and SPSS 22.0 (SPSS Inc. Chicago, IL, USA) to perform the statistical analyses.

## Results

### Patient Characteristics

**Figure 1:**
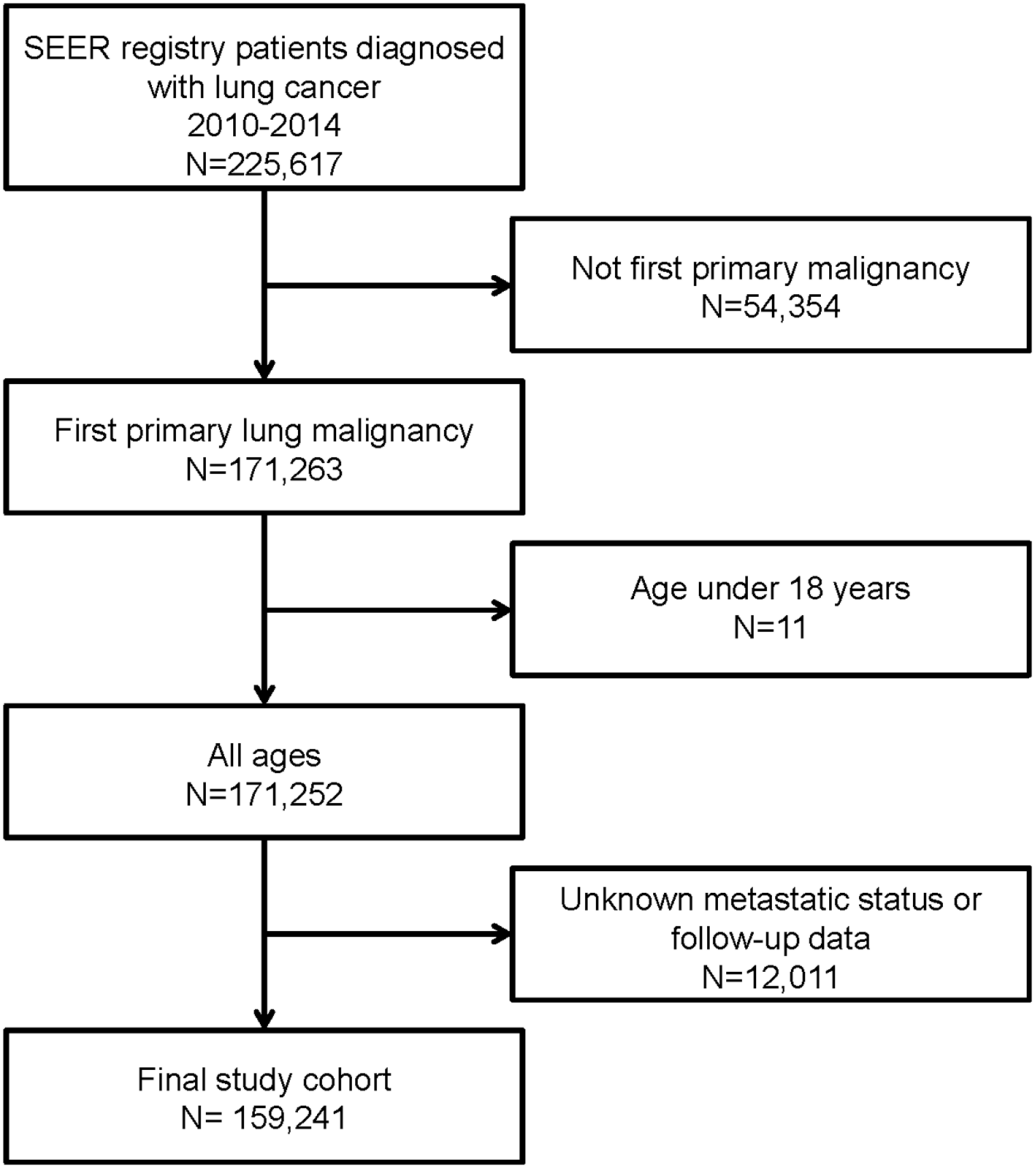
Flowchart of patient selection in this study.

According to the inclusion and exclusion criteria, we finally enrolled 159,241 cases diagnosed with lung cancer. Detailed selection flowchart was illustrated in Figure 1. Among the final cohort, 75,231 cases (47.2%) were adenocarcinoma, 37,179 cases (23.3%) were squamous cell carcinoma, 2,832 cases (1.8%) were large-cell lung cancer, 22,709 cases (14.3%) were small -cell lung cancer, and 21,290 cases (13.4%) were non-small cell lung cancer. The baseline demographic and clinicopathological parameters according to different metastatic lesions were shown in Table 1.

**Table 1:** Baseline clinical characteristics of lung cancer patients in SEER database.

**Characteristics**	**Bone metastasis**			**Brain metastasis**			**Liver metastasis**			**DL metastasis**		
	**No (%)**	**Yes (%)**	***P***	**No (%)**	**Yes (%)**	***P***	**No (%)**	**Yes (%)**	***P***	**No (%)**	**Yes (%)**	***P***
**Histological type**												
Adenocarcinoma	59,090 (78.5)	16,141 (21.5)	<0.001	63,943 (85.0)	11,288 (15.0)	<0.001	68,647 (91.2)	6,584 (8.8)	<0.001	70,399 (93.6)	4,832 (6.4)	<0.001
Squamous cell carcinoma	33,033 (88.8)	4,146 (11.2)		35,061 (94.3)	2,118 (5.7)		34,874 (93.8)	2,305 (6.2)		35,737 (88.0)	1,442 (12.0)	
LCLC	2,322 (82.0)	510 (18.0)		2,342 (82.7)	490 (17.3)		2,441 (86.2)	391 (13.8)		2,601 (91.8)	231 (8.2)	
SCLC	17,427 (76.7)	5,282 (23.3)		18.913 (83.3)	3,796 (16.7)		15,549 (68.5)	7,160 (31.5)		20,308 (89.4)	2,401 (10.6)	
NSCLC/NOS	16,749 (78.7)	4,541 (21.3)		17,736 (83.3)	3,554 (16.7)		18,341 (86.1)	2,949 (13.9)		19,803 (93.0)	1,487 (7.0)	
**Gender**												
Male	66,188 (79.2)	17,382 (20.8)	<0.001	72,491 (86.7)	11,079 (13.3)	0.295	72,929 (87.3)	10,641 (12.7)	<0.001	77,683 (93.0)	5,887 (7.0)	<0.001
Female	62,433 (82.5)	13,238 (17.5)		65,504 (86.6)	10,167 (13.4)		66,923 (88.4)	8,748 (11.6)		71,165 (94.0)	4,506 (6.0)	
**Age**												
<50	5,129 (74.9)	1,717 (25.1)	<0.001	5,361 (78.3)	1,485 (21.7)	<0.001	5,936 (86.7)	910 (13.3)	<0.001	6,156 (89.9)	690 (10.1)	<0.001
51–65	39,240 (77.7)	11,276 (22.3)		41,365 (81.9)	9,151 (18.1)		43,568 (86.2)	6,948 (13.8)		46,254 (91.6)	4,262 (8.4)	
≥65	84,252 (82.7)	17,627 (17.3)		91,269 (89.6)	10610 (10.4)		90,348 (88.7)	11,531 (11.3)		96,438 (94.7)	5,441 (5.3)	
**Marital status**												
Married	62,526 (79.8)	15,860 (20.2)	<0.001	67,634 (86.3)	10,752 (13.7)	<0.001	68,786 (87.8)	9,600 (12.2)	0.625	73,147 (93.3)	5,239 (6.7)	0.035
Unmarried	60,049 (81.7)	13,420 (18.3)		63,833 (86.9)	9,636 (13.1)		64,562 (87.9)	8,907 (12.1)		68,772 (93.6)	4,697 (6.4)	
Unknown	6,046 (81.9)	1,340 (18.1)		6,528 (88.4)	858 (11.6)		6,504 (88.1)	882 (11.9)		6,929 (93.8)	457 (6.2)	
**Race**												
White	103,885 (80.9)	24,502 (19.1)	<0.001	111,722 (87.0)	16,665 (13.0)	<0.001	112,357 (87.5)	16,030 (12.5)	<0.001	120,108 (93.6)	8,279 (6.4)	0.009
Black	15,612 (81.5)	3,555 (18.5)		16,447 (85.8)	2,720 (14.2)		17,055 (89.0)	2,112 (11.0)		17,889 (93.3)	1,278 (6.7)	
Others	9,124 (78.1)	2,563 (21.9)		9,826 (84.1)	1,861 (15.9)		10,440 (89.3)	1,247 (10.7)		10,851 (92.8)	836 (7.2)	
**Grade**												
I	7,240 (94.3)	434 (5.7)	<0.001	7,405 (96.5)	269 (3.5)	<0.001	7,521 (98.0)	153 (2.0)	<0.001	7,573 (98.7)	101 (1.3)	<0.001
II	23,464 (90.6)	2,444 (9.4)		24,237 (93.6)	1,671 (6.4)		25,041 (96.7)	867 (3.3)		25,312 (97.7)	596 (2.3)	
III	33,521 (83.9)	6,441 (16.1)		34,842 (87.2)	5,120 (12.8)		36,528 (91.4)	3,434 (8.6)		37,754 (94.5)	2,208 (5.5)	
IV	3,901 (80.4)	951 (19.6)		4,121 (84.9)	731 (15.1)		3,836 (79.1)	1,016 (20.9)		4,434 (91.4)	418 (8.6)	
Unknown	60,495 (74.8)	20,350 (25.2)		67,390 (83.4)	13,455 (16.6)		66,926 (82.8)	13,919 (17.2)		73,775 (91.3)	7,070 (8.7)	
**Size (cm)**												
<2.0	17,612 (89.1)	2,160 (10.9)	<0.001	18,206 (92.1)	1,566 (7.9)	<0.001	18,536 (93.7)	1,236 (6.3)	<0.001	19,025 (96.2)	747 (3.8)	<0.001
2.0–4.9	51,049 (82.2)	11,029 (17.8)		54,351 (87.6)	7,727 (12.4)		55,967 (90.2)	6,111 (9.8)		58,838 (94.8)	3,240 (5.2)	
5.0–9.9	32,257 (79.1)	8,511 (20.9)		34,305 (84.1)	6,463 (15.9)		35,269 (86.5)	5,499 (13.5)		37,835 (92.8)	2,933 (7.2)	
≥10.0	3,844 (79.8)	974 (20.2)		4,067 (85.4)	751 (15.6)		4,123 (85.6)	695 (14.4)		4,383 (91.0)	435 (9.0)	
Unknown	23,859 (75.0)	7,946 (25.0)		27,066 (85.1)	4,739 (14.9)		25,957 (81.6)	5,848 (18.4)		28,767 (90.4)	3,038 (9.6)	
**Regional lymph node invasion**												
N0	53,156 (90.4)	5,630 (9.6)	<0.001	54,430 (92.6)	4,356 (7.4)	<0.001	55,846 (95.0)	2,940 (5.0)	<0.001	57,998 (98.7)	788 (1.3)	<0.001
N1	10,999 (82.5)	2,337 (17.5)		11,604 (87.0)	1,732 (13.0)		11,952 (89.6)	1,384 (10.4)		12,824 (96.2)	512 (3.8)	
N2	44,427 (75.6)	14,321 (24.4)		48,970 (83.4)	9,778 (16.6)		48,861 (83.2)	9,887 (16.8)		54,392 (92.6)	4,356 (7.4)	
N3	15,408 (70.4)	6,479 (29.6)		17,707 (80.9)	4,180 (19.1)		18,009 (82.3)	3,878 (17.7)		17,249 (79.6)	4,458 (20.4)	
NX	4,631 (71.4)	1,853 (28.6)		5,284 (81.5)	1,200 (18.5)		5,184 (80.0)	1,300 (20.0)		6,205 (95.7)	279 (4.3)	
**Surgery**												
Yes	32,518 (98.8)	400 (1.2)	<0.001	32,346 (98.3)	572 (1.7)	<0.001	32,760 (99.5)	158 (0.5)	<0.001	32,783 (99.6)	135 (0.4)	<0.001
No	96,103 (76.1)	30,220 (23.9)		105,649 (83.6)	20,674 (16.4)		107,092 (84.8)	19,231 (15.2)		116,065 (91.9)	10,258 (8.1)	
**Chemotherapy**												
Yes	58,803 (77.5)	17,117 (22.5)	<0.001	64,268 (84.7)	11,652 (15.3)	<0.001	65,583 (86.4)	10,337 (13.6)	<0.001	69,507 (91.6)	6,413 (8.4)	<0.001
No	69,818 (83.8)	13,503 (16.2)		73,727 (88.5)	9,594 (11.5)		74,269 (89.1)	9,052 (10.9)		79,341 (95.2)	3,980 (4.8)	
**Radiation therapy**												
Yes	50,966 (76.8)	15,387 (23.2)	<0.001	50,744 (76.5)	15,609 (23.5)	<0.001	59,939 (90.3)	6,414 (9.7)	<0.001	62,016 (93.5)	4,337 (6.5)	0.895
No	77,655 (83.6)	15,233 (16.4)		87,251 (93.9)	5,637 (6.1)		79,913 (86.0)	12,975 (14.0)		86,832 (93.5)	6,056 (6.5)	

*ΔOthers include American Indian, AK Native, Asian, and Pacific Islander. LCLC, Large-cell lung cancer; SCLC, Small-cell lung cancer; NSCLC, Non-small cell lung cancer*.

Among the final cohort, 60,580 cases (38.0%) were recorded as extrathoracic metastasis. In total, the four metastatic lesions (bone, brain, liver, and distant lymph node) accounted for 94.0% (56,933/60,580) of all extrathoracic metastatic sites. And the frequencies of bone, brain, liver and distant lymph node (DL) metastasis were 19.2% (30,620/159,241), 13.3% (21,246/159,241), 12.2% (19,380/159,241), and 6.5% (10,393/159,241), respectively.

### Metastatic Pattern

**Figure 2:**
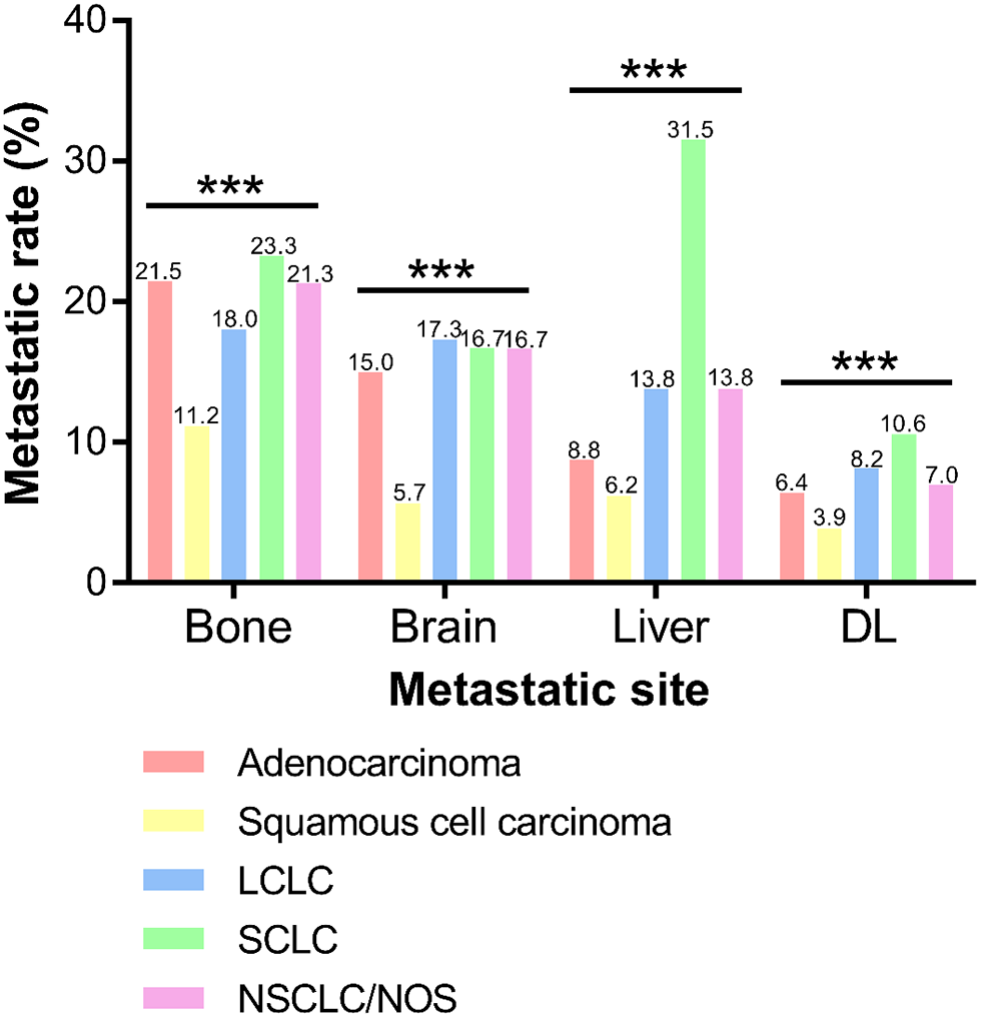
Frequencies of extrathoracic metastatic organs according to different histological types. DL, distant lymph node. (^***^P < 0.001).

As shown in Figure 2, incidence rate of bone metastasis was the highest in SCLC (23.3%), followed by adenocarcinoma (21.5%), NSCLC/NOS (21.3%), LCLC (18.0%), and squamous cell carcinoma (11.2%). And frequencies of brain metastasis were 15.0, 5.7, 17.3, 16.7 and 16.7% in adenocarcinoma, squamous cell carcinoma, LCLC, SCLC, and NSCLC/NOS, respectively. The incidence of brain metasiasis almost the same except squamous cell carcinoma. Also, the metastatic rate of liver was extremely high in SCLC (31.5%) and relatively low in squamous cell carcinoma (6.2%). In addition, the frequency of DL metastasis in SCLC (10.6%) was higher than LCLC (8.2%), NSCLC/NOS (7.0%), adenocarcinoma (6.4%) and squamous cell carcinoma (3.9%).

For clinicopathological features, metastatic group tended to have younger age, poorer tumor differentiation, larger tumor size and higher frequency of regional lymph node invasion (Table 1). As for therapies, advanced-stage patients received less surgery and more chemotherapy than non-metastatic patients. And patients with bone or brain metastasis received more radiation therapy than non-metastatic patients.

### Combination of Metastases

**Figure 3:**
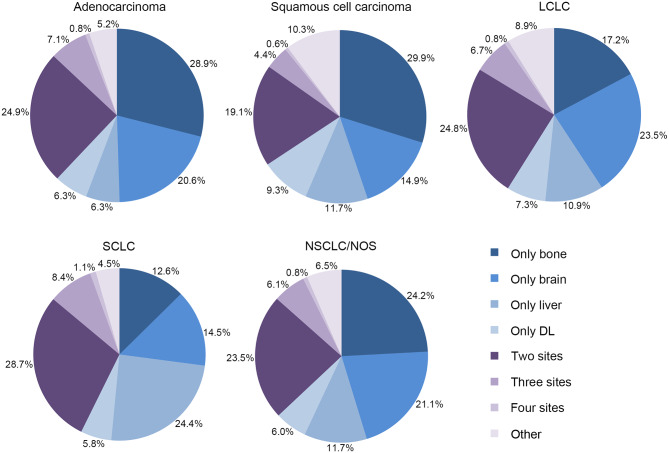
Relative rates of single and combined metastatic sites in different histological types.

For further analyzing combination of metastases, we performed pie charts to investigate single-metastases and co-metastases among different histological types of lung cancer (Figure 3). It is shown that bone was the leading lesion as a single metastatic site in adenocarcinoma (28.9%), squamous cell carcinoma (29.9%) and NSCLC/NOS (24.2%). Also, brain was the leading single-metastatic lesion in LCLC (23.5%), and liver was the most frequent site in SCLC (24.4%). As for combination of metastases, bi-site pattern (adenocarcinoma: 24.9%, squamous cell carcinoma: 19.1%, LCLC: 24.8%, SCLC: 28.7%, and NSCLC/NOS: 23.5%) was significantly higher than tri-site (adenocarcinoma: 7.1%, squamous cell carcinoma: 4.4%, LCLC: 6.7%, SCLC: 8.4%, and NSCLC/NOS: 6.1%) and tetra-site pattern (Adenocarcinoma: 0.8%, Squamous cell carcinoma: 0.6%, LCLC: 0.8%, SCLC: 1.1%, and NSCLC/NOS: 0.8%).

**Figure 4:**
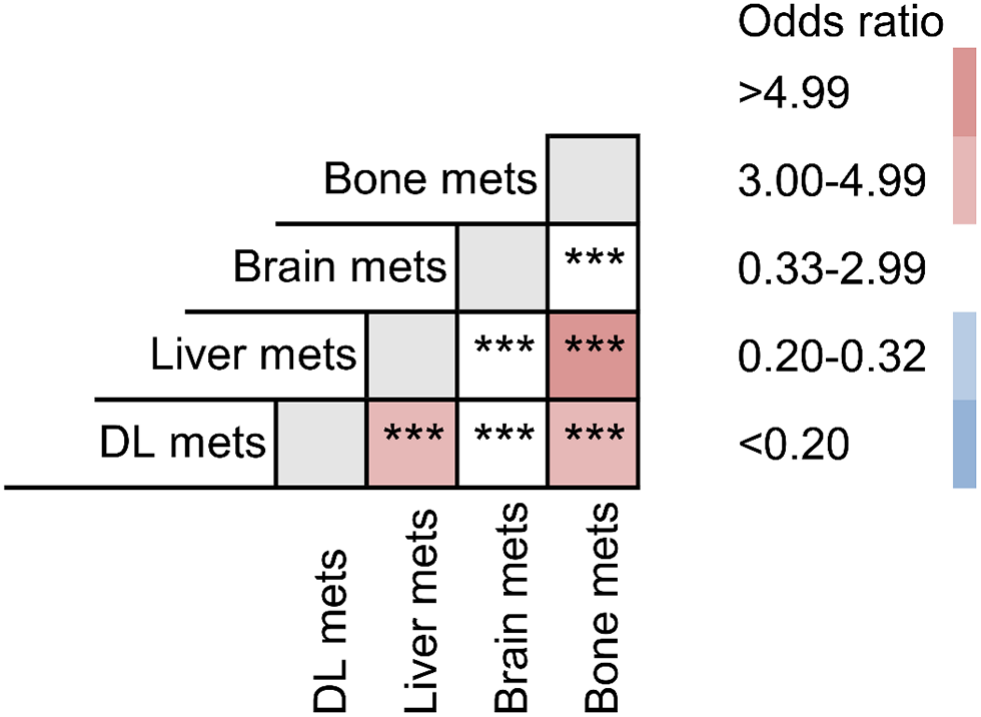
Odds ratio comparison among different metastatic combinations. ^***^P < 0.001.

Furthermore, we calculated odds ratios to compare each possible combination of different extrathoracic metastatic lesions (Figure 4, Supplementary Figure 1). Bone preferentially tended to co-metastasize with liver (OR: 5.287) and DL (OR: 3.013). And liver metastasis was significantly correlated with DL metastasis (OR: 3.093).

### Survival

**Figure 5:**
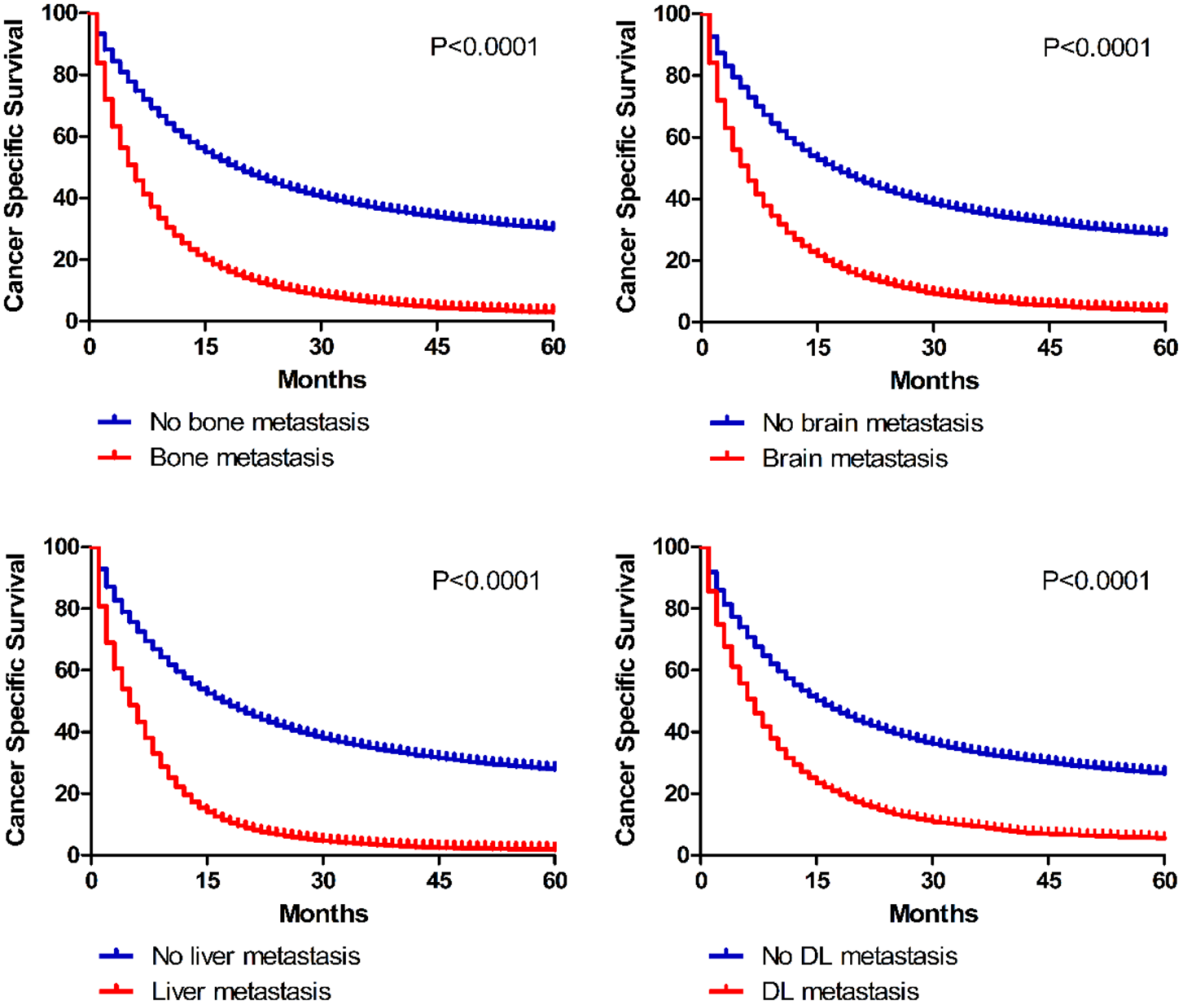
Kaplan–Meier curves of cancer specific survival in patients according to metastatic status.

In the present study, we analyzed 1-year OS and CSS in cases with diverse extrathoracic metastatic lesions (Table 2). Univariate analyses indicated that survival differences existed between non-metastatic and metastatic patients (OS: bone 50.8 vs. 20.0%, brain 48.4 vs. 22.2%, liver 49.2 vs. 13.6%, DL 46.4 vs. 22.9%; CSS: bone 57.5 vs. 25.9%, brain 55.1 vs. 27.4%, liver 55.8 vs. 20.1%, DL 53.0 vs. 28.8%). And Kaplan–Meier curves further illustrated the survival data between non-metastatic and metastatic groups (Figure 5).

**Table 2:** Survival analysis in diverse metastatic organs.

**Parameter**	**1-year OS (%)**	**Univariate analysis**		**1-year CSS (%)**	**Univariate analysis**	
		**Log rank χ^2^ test**	***P***		**Log rank χ^2^ test**	***P***
**Bone**						
No metastasis	50.8	12615.144	<0.001	57.5	13549.160	<0.001
Metastasis	20.0			25.9		
**Brain**						
No metastasis	48.4	6499.456	<0.001	55.1	7316.606	<0.001
Metastasis	22.1			27.4		
**Liver**						
No metastasis	49.2	14245.964	<0.001	55.8	14802.929	<0.001
Metastasis	13.6			20.1		
**Distant lymph nodes**						
No metastasis	46.4	2748.753	<0.001	53.0	2990.572	<0.001
Metastasis	22.9			28.8		

*OS, Overall Survival; CSS, Cancer Specific Survival*.

Furthermore, Cox regression models were conducted to identify independent prognostic factors (Table 3). With adjusting for histological type, gender, age, race, marital status, grade, tumor size, regional lymph node invasion and therapies, all extrathoracic metastatic lesions were independent risk factors for OS (bone: HR 1.312, 95%CI 1.302–1.321; brain: HR 1.339, 95%CI 1.328–1.351; liver: HR 1.344, 95%CI 1.333–1.355; DL: HR 1.263, 95%CI 1.235–1.290) and CSS (bone: HR 1.337, 95%CI 1.328–1.348; brain: HR 1.368, 95%CI 1.357–1.381; liver: HR 1.375, 95%CI 1.363–1.388; DL: HR 1.283, 95%CI 1.254–1.313).

**Table 3:** Multivariate analyses of overall and cancer-specific survival in related to metastatic sites.

**Variable**	**Overall survival**		**Cancer-specific survival**	
	**HR (95% CI)**	***P***	**HR (95% CI)**	***P***
No metastasis	Reference		Reference	
Bone metastasis	1.312 (1.302–1.321)	<0.001	1.337 (1.328–1.348)	<0.001
Brain metastasis	1.339 (1.328–1.351)	<0.001	1.368 (1.357–1.381)	<0.001
Liver metastasis	1.344 (1.333–1.355)	<0.001	1.375 (1.363–1.388)	<0.001
DL metastasis	1.263 (1.235–1.290)	<0.001	1.283 (1.254–1.313)	<0.001

*Adjusted for histological type, gender, age, race, marital status, grade, tumor size, regional lymph node invasion and therapies. OS, Overall Survival; CSS, Cancer Specific Survival; HR, Hazard Ratio*.

**Figure 6:**
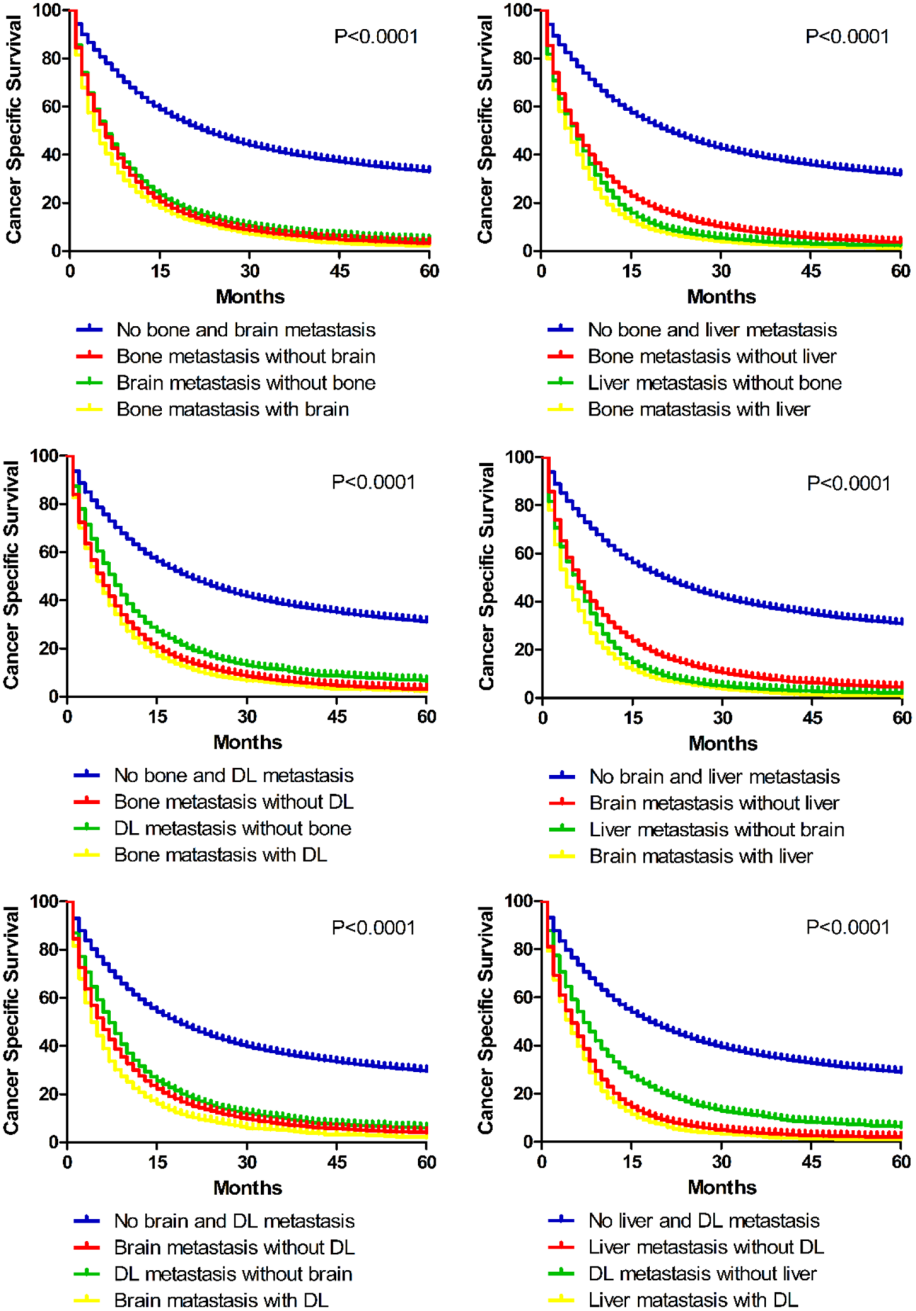
Kaplan–Meier curves of cancer specific survival in patients with different bi-site metastatic patterns.

Additionally, survival differences between different bi-organ metastases were analyzed (Figure 6). It is suggested in the Kaplan–Meier curves that combined metastasis resulted in worse prognostic ending than the separated single-organ metastasis. Once metastasis happens, lung cancer patients might get a worse outcome.

### Discussion

Lung cancer related deaths are mainly attributable to extrathoracic metastasis (16, 17). Advanced lung cancer seems to metastasize to lymph nodes and other distant organs, such as brain, bone and liver. Most metastasis could cause corresponding symptoms which is represented by the N and M staging in the TNM system. In order to understand its metastatic patterns, we elucidated the following points in this research: (1) Comparing the frequencies of different metastatic lesions in different histological types; (2) Elaborating the tendency of combined metastases; (3) Clarifying the prognostic significance of single-site and bi-site metastases. As the first comprehensive, population-based research focusing on metastatic patterns in different histological types of lung cancer, the findings may provide sufficient information for clinical decision and cancer research.

According to the reported data, bone and brain were two leading distant targets for metastasis in NSCLC (18, 19). Our results further supported these findings, suggesting that bone was the most common metastatic site, followed by brain, liver and DL in all histological sites of NSCLC (including adenocarcinoma, squamous cell carcinoma, LCLC and NSCLC/NOS). By comparing the frequency of extrathoracic metastasis in diverse histological types of NSCLC, we found that more than 30% of adenocarcinoma and LCLC patients showed distant metastasis, while squamous cell cancer had the lowest rate of distal metastasis. Moreover, among all histological types of lung cancer, SCLC had the highest frequency of extrathoracic metastasis, especially to the liver, which is consistent with the reported data in previous studies (20–22). So, according to these conclusion, adenocarcinoma and LCLC patients could be arranged serious and continual follow-up, more importantly, these patients could take cutting-edge therapies, such as combined immunotherapy, neoadjuvant chemotherapy, and so on. For SCLC patients, liver ultrasound and CT scan need to be focused on.

Notably, according to the clinicopathological features, metastatic group tended to have a poorer tumor differentiation, a larger tumor size and a higher rate of regional lymph node invasion, which indicated a more aggressive and invasive hallmark of tumor biology. Compared to non-metastatic patients, advanced-stage patients received less surgery and more chemotherapy, because they lost the chance of curative resection at the time of diagnosis. And since radiation could control tumor growth of metastatic nodules as well as alleviating symptoms, patients with bone or brain metastasis received more radiation therapy than non-metastatic patients.

But these conclusions have their own historical limitaions. With the development of immunotherapy and neoadjuvant chemotherapy, patients may benefit from these modern and fancy therapies, and they could even get the chance of surgery due to the shrinking tumors. Considering these demographic, clinicopathological and treatment variables that may have impact on survival outcomes, we further conducted multivariate analysis and found that all single-site metastases were independent prognostic factors.

To our knowledge, no previous population-based researches studied the combined metastatic patterns of lung cancer. Our results indicated that bone preferentially tended to co-metastasize with liver and distal lymph nodes. And liver metastasis was significantly correlated with distant lymph node metastasis. To our knowledge, analyzing tendency of co-metastases would be rather useful to assess potential risks and make diagnosis and treatment strategies. Once bone metastasis was found, we need to screen the liver and get an enhanced CT to detect the lymph nodes. Thus, patients may get a comprehensive system treatment. And, if liver metastasis needed to be surgical removed, doctors should note that lymph node dissection is the necessary and best choice. Moreover, we further assessed the prognostic values of bi-site metastases. As shown in Kaplan–Meier curves, combined metastasis resulted in worse prognostic ending than the separated single-organ metastasis. So patients with multi-organ metastasis may need more aggressive therapeutic regimens.

Though we seriously performed this population-based research, there may still be several potential limitations. The first limitation may be the retrospective nature of this study. We only enrolled patients with detailed distal metastasis since SEER database recorded from year 2010. Second, information of extrathoracic metastatic sites was restricted to bone, brain, liver, and DL. However, these four metastatic lesions accounted for the majority of extrathoracic metastatic sites in lung cancer. Third, the metastasis condition from SEER was synchronous when diagnosed, but in the real world, metachronous carcinoma accounts for the majority. These limitations could cause bias in some results.

In a word, we comprehensively analyzed the pattern of extrathoracic metastases in different histological types of lung cancer in this population-based study. We found that the frequency of bone metastasis was the highest in adenocarcinoma, squamous cell carcinoma, LCLC and NSCLC/NOS, while liver was the most common metastatic site in SCLC. Bi-site metastases occurred more common than tri-site and tetra-site metastases. Several metastatic sites, such as bone and liver, intended to co-metastasize preferentially. All single-site metastases were independent prognostic factors and co-metastases ended up with even worse survival outcomes. Thus, our findings would be beneficial for future research design and clinical practice.

## Data Availability Statement

The datasets generated for this study are available on request to the corresponding author.

## Author Contributions

Conception and design: XW and ZW. Development of methodology: XW, ZW, and JP. Acquisition of data, analysis and interpretation of data (e.g., statistical analysis, biostatistics, computational analysis): Z-YL, DX, H-JZ, and S-HW. Writing, review and/or revision of the manuscript: XW, ZW, and JP. Study supervision: D-YH and X-FC. All authors reviewed and approved the final manuscript.

## Conflict of Interest

The authors declare that the research was conducted in the absence of any commercial or financial relationships that could be construed as a potential conflict of interest.

## Supplementary Material

Supplementary Figure 1 The number of odds ratio comparison among different metastatic combinations.

## References

[1] Siegel RL Miller KD Jemal A Cancer statistics, 2019. CA Cancer J Clin. (2019) 69:7–34. 10.3322/caac.21551 30620402

[2] Ferlay J Soerjomataram I Dikshit R Eser S Mathers C Rebelo M. Cancer incidence and mortality worldwide: sources, methods and major patterns in GLOBOCAN 2012. Int J Cancer. (2015) 136:E359–86. 10.1002/ijc.29210 25220842

[3] de Groot PM Carter BW Betancourt CS Erasmus JJ. Staging of lung cancer. Clin Chest Med. (2015) 36:179–96. 10.1016/j.ccm.2015.02.004 26024599

[4] Woodard GA Jones KD Jablons DM. Lung cancer staging and prognosis. Cancer Treat Res. (2016) 170:47–75. 10.1007/978-3-319-40389-2_3 27535389

[5] Hirsch FR Scagliotti GV Mulshine JL Kwon R Curran WJ Wu YL. Lung cancer: current therapies and new targeted treatments. Lancet. (2017) 389:299–311. 10.1016/S0140-6736(16)30958-8 27574741

[6] Mayekar MK Bivona TG. Current landscape of targeted therapy in lung cancer. Clin Pharmocol Ther. (2017) 102:757–64. 10.1002/cpt.810 28786099

[7] Tsao AS Scagliotti GV Bunn PJ Carbone DP Warren GW Bai C. Scientific advances in lung cancer 2015. J Thorac Oncol. (2016) 11:613–38. 10.1016/j.jtho.2016.03.012 27013409

[8] Rodriguez-Canales J Parra-Cuentas E Wistuba II. Diagnosis and molecular classification of lung cancer. Cancer Treat Res. (2016) 170:25–46. 10.1007/978-3-319-40389-2_2 27535388

[9] Goldstraw P Ball D Jett JR Le Chevalier T Lim E Nicholson AG. Non-small-cell lung cancer. Lancet. (2011) 378:1727–40. 10.1016/S0140-6736(10)62101-0 21565398

[10] Rudin CM Ismaila N Hann CL Malhotra N Movsas B Norris K. Treatment of small-cell lung cancer: american society of clinical oncology endorsement of the american college of chest physicians guideline. J Clin Oncol. (2015) 33:4106–11. 10.1200/JCO.2015.63.7918 26351333

[11] van Meerbeeck JP Fennell DA De Ruysscher DK. Small-cell lung cancer. Lancet. (2011) 378:1741–55. 10.1016/S0140-6736(11)60165-7 21565397

[12] Goldstraw P Chansky K Crowley J Rami-Porta R Asamura H Eberhardt WE. The IASLC lung cancer staging project: proposals for revision of the TNM stage groupings in the forthcoming (eighth) edition of the TNM classification for lung cancer. J Thorac Oncol. (2016) 11:39–51. 10.1016/j.jtho.2015.09.009 26762738

[13] Sanchez DCEJ Abal AJ Melchor IR Miravet SL Nunez AA Hernandez HJ Tumor, node and metastasis classification of lung cancer–M1a versus M1b–analysis of M descriptors and other prognostic factors. Lung Cancer. (2014) 84:182–9. 10.1016/j.lungcan.2014.02.006 24629637

[14] He YY Zhang XC Yang JJ Niu FY Zeng Z Yan HH. Prognostic significance of genotype and number of metastatic sites in advanced non-small-cell lung cancer. Clin Lung Cancer. (2014) 15:441–7. 10.1016/j.cllc.2014.06.006 25044104

[15] Eberhardt WE Mitchell A Crowley J Kondo H Kim YT Turrisi AR. The IASLC lung cancer staging project: proposals for the revision of the m descriptors in the forthcoming eighth edition of the TNM classification of lung cancer. J Thorac Oncol. (2015) 10:1515–22. 10.1097/JTO.0000000000000673 26536193

[16] Shroff GS Viswanathan C Carter BW Benveniste MF Truong MT Sabloff BS. Staging lung cancer: metastasis. Radiol Clin North Am. (2018) 56:411–8. 10.1016/j.rcl.2018.01.009 29622076

[17] Bates JE Milano MT. Prognostic significance of sites of extrathoracic metastasis in patients with non-small cell lung cancer. J Thorac Dis. (2017) 9:1903–10. 10.21037/jtd.2017.06.117 28839988 PMC5543004

[18] Hattori A Takamochi K Oh S Suzuki K. New revisions and current issues in the eighth edition of the TNM classification for non-small cell lung cancer. Jpn J Clin Oncol. (2019) 49:3–11. 10.1093/jjco/hyy142 30277521

[19] Franchino F Ruda R Soffietti R. Mechanisms and therapy for cancer metastasis to the brain. Front Oncol. (2018) 8:161. 10.3389/fonc.2018.00161 29881714 PMC5976742

[20] Cai H Wang H Li Z Lin J Yu J. The prognostic analysis of different metastatic patterns in extensive-stage small-cell lung cancer patients: a large population-based study. Future Oncol. (2018) 14:1397–407. 10.2217/fon-2017-0706 29359568

[21] Nakazawa K Kurishima K Tamura T Kagohashi K Ishikawa H Satoh H. Specific organ metastases and survival in small cell lung cancer. Oncol Lett. (2012) 4:617–20. 10.3892/ol.2012.792 23205072 PMC3506697

[22] Byers LA Rudin CM. Small cell lung cancer: where do we go from here? Cancer Am Cancer Soc. (2015) 121:664–72. 10.1002/cncr.29098 25336398 PMC5497465

