# Supplementary figures and images for: Patterns of Extrathoracic Metastases in Different Histological Types of Lung Cancer
Source: Front Oncol. 2020 May 19;10:715. doi: 10.3389/fonc.2020.00715 (PMC7248315; doi:10.3389/fonc.2020.00715)

|         |            |       |            |       |
|---------|------------|-------|------------|-------|
|         |            |       | Bone mets  |       |
|         |            |       | Brain mets | 2.423 |
|         | Liver mets |       | 2.11       | 5.287 |
| DL mets |            | 3.093 | 2.185      | 3.013 |

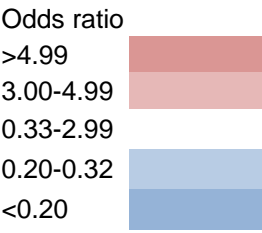

Supplement: Supplementary Figure 1 — The number of odds ratio comparison among different metastatic combinations. [file Data_Sheet_1.PDF]
